# Nutritional case-control study of Calcium Nephrolithiasis

**DOI:** 10.1186/1753-6561-6-S3-P46

**Published:** 2012-06-01

**Authors:** Laura Soldati, Elena Dogliotti, Annalisa Terranegra, Tiziana Meschi, Antonio Nouvenne, Beatrice Prati, Giuseppe Vezzoli, Loris Borghi

**Affiliations:** 1 Dep. Medicine, Surgery, Dentistry, San Paolo Hospital, University of Milan, Milan, 20100, Italy; 2 Nephrology and Dialysis Unit, San Raffaele Hospital, Milan, 20100, Italy; 3 Dep. Clinical Sciences, University of Parma, Parma, 43121, Italy

## Background

It is well established that nutritional habits are relevant in the prevention of Idiopathic Calcium Nephrolithiasis (ICN) and the Mediterranean diet is believed to be protective against nephrolithiasis and not only against cardiovascular events. A case-control study was performed to establish the nutritional habits of Italian ICN patients and the nutritional determinants of lithogenic risk in the considered population.

## Materials and methods

Calcium stone formers (SF: n=232, 145 F and 87 M, age 42.25±10.70 yrs, BMI 23.95±3.98 kg/m^2^) and controls (CTR: n=259, 220 F and 39 M, age 40.97±10.73 yrs, BMI 23.38±3.73 kg/m^2^) were enrolled. A 3-day nutritional diary was analyzed by the software Dietosystem (DS Medica, Milano, Italy). The nutritional intake was also compared to the Italian nutritional guidelines. Urinary factors were analyzed from 24h urine collection and statistical analysis was performed by the SPSS software.

## Results

Urinary data showed an increased excretion of Ca^2+^ (5.55±2.70mg/24h vs 4.12±1.98mg/24h, p<0.05) and a decreased excretion of K^+^ (49.36±17.81mmol/24h vs 54.55±17.2mmol/24h, p<0.05) and citrate (544.29±262.82mg/24h vs 660.09±247.21mg/24h, p<0.05) in SF than CTR. Nutritional analysis found differences between SF and CTR: a higher caloric intake (2013.03±753.77 Kcal/ die vs 1933.39±502.20 Kcal/die, p<0.05), a higher total protein intake (79.8±22.70 g/die vs 75.6±21.31 g/die, p<0.05) and a higher vegetable protein intake (25.74±9.41 g/die vs 22.94±8.73 g/die, p<0.05) were observed in SF than CTR. Moreover SF showed a higher intake of sodium, oxalate, complex carbohydrates, purines, arachidonic acid and a higher acid load. The comparison of SF nutritional intake to the Italian guidelines demonstrated a prevalence of hyperproteic, hyperlipidic and hypercaloric diet, a low calcium, potassium and fiber intake, and a high salt, phosphorus and oxalate intake. Water intake covered the recommended quantity (1.5-2 L/die) for healthy population, but not for SF (>2 L/die).

## Conclusions

In conclusion, our study found different nutritional habits between SF and CTR and confirmed some dietetic errors respect to national dietetic recommendations. These errors could increase the risk to develop ICN in subjects with a lithogenic genetic background and confirm the usefulness to give nutritional advices to SF patients.

